# 1,3-Bis[(5-amino­furan-2-yl)meth­yl]-3,4,5,6-tetra­hydro­pyrimidin-1-ium hexa­fluoro­phosphate

**DOI:** 10.1107/S1600536813020187

**Published:** 2013-07-27

**Authors:** Mehmet Akkurt, Senem Akkoç, Yetkin Gök, Muhammad Nawaz Tahir

**Affiliations:** a Department of Physics, Faculty of Sciences, Erciyes University, 38039 Kayseri, Turkey; b Department of Chemistry, Faculty of Sciences, Erciyes University, 38039 Kayseri, Turkey; c Department of Chemistry, Faculty of Arts and Sciences, Inönü University, 44280, Malatya, Turkey; d Department of Physics, University of Sargodha, Sargodha, Pakistan

## Abstract

The asymmetric unit of the title salt, C_16_H_21_N_2_O_2_^+^·PF_6_^−^, contains half of the whole ion pair, which has crystallographic mirror symmetry. Two F atoms related by the mirror plane are disordered over two sites of equal occupancy. The dihedral angle between the central ring and the furan ring is 59.3 ()°. In the crystal, the anions and cations are linked through C—H⋯F inter­actions, forming a three-dimensional network.

## Related literature

*N*-heterocyclic carbene (NHC)-metal complexes have attracted much attention, particularly for their functions in catalytic reactions, see: Akkoç & Gök (2013 ▶); Arduengo *et al.* (1992 ▶); Bagherzadeh *et al.* (2012 ▶); Hermann (2002 ▶); Lee *et al.* (2013 ▶); Saba *et al.* (1991 ▶); Yiğit *et al.* (2007 ▶); Özdemir *et al.* (2001 ▶); Çetinkaya *et al.* (1997 ▶). For bond-length data, see: Allen *et al.* (1987 ▶).
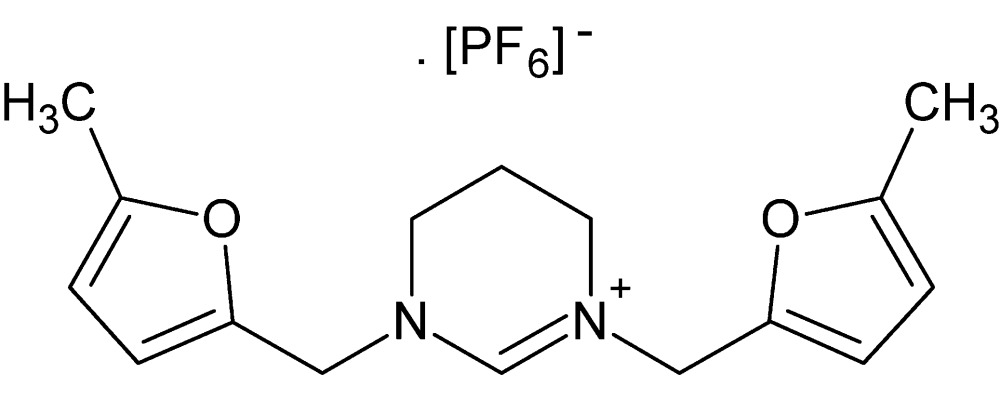


## Experimental

### 

#### Crystal data

C_16_H_21_N_2_O_2_^+^·PF_6_^−^
*M* *_r_* = 418.32
Monoclinic, 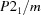

*a* = 6.0793 (6) Å
*b* = 18.879 (2) Å
*c* = 8.5750 (9) Å
β = 100.747 (5)°
*V* = 966.90 (17) Å^3^
*Z* = 2
Mo *K*α radiation
μ = 0.21 mm^−1^
*T* = 296 K
0.35 × 0.28 × 0.23 mm

#### Data collection

Bruker Kappa APEXII CCD diffractometer
Absorption correction: part of the refinement model (Δ*F*) (*XABS2*; Parkin *et al.*, 1995 ▶) *T*_min_ = 0.932, *T*_max_ = 0.953
1677 measured reflections
1677 independent reflections
1089 reflections with *I* > 2σ(*I*)
*R*_int_ = 0.000

#### Refinement

*R*[*F* ^2^ > 2σ(*F* ^2^)] = 0.077
*wR*(*F* ^2^) = 0.247
*S* = 1.08
1677 reflections
144 parameters
H-atom parameters constrained
Δρ_max_ = 0.36 e Å^−3^
Δρ_min_ = −0.55 e Å^−3^

### 

Data collection: *APEX2* (Bruker, 2009 ▶); cell refinement: *SAINT* (Bruker, 2009 ▶); data reduction: *SAINT*; program(s) used to solve structure: *SHELXS97* (Sheldrick, 2008 ▶); program(s) used to refine structure: *SHELXL97* (Sheldrick, 2008 ▶); molecular graphics: *ORTEP-3 for Windows* (Farrugia, 2012 ▶) and *PLATON* (Spek, 2009 ▶); software used to prepare material for publication: *WinGX* (Farrugia, 2012 ▶) and *PLATON*.

## Supplementary Material

Crystal structure: contains datablock(s) global, I. DOI: 10.1107/S1600536813020187/hg5332sup1.cif

Structure factors: contains datablock(s) I. DOI: 10.1107/S1600536813020187/hg5332Isup2.hkl

Supplementary material file. DOI: 10.1107/S1600536813020187/hg5332Isup3.cml

Additional supplementary materials:  crystallographic information; 3D view; checkCIF report

## Figures and Tables

**Table 1:** Hydrogen-bond geometry (Å, °)

*D*—H⋯*A*	*D*—H	H⋯*A*	*D*⋯*A*	*D*—H⋯*A*
C1—H1⋯F2^i^	0.93	2.40	3.312 (8)	167
C1—H1⋯F4^ii^	0.93	2.52	3.044 (8)	116

Symmetry codes: (i) 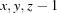; (ii) 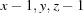.

**Fig. 1.**
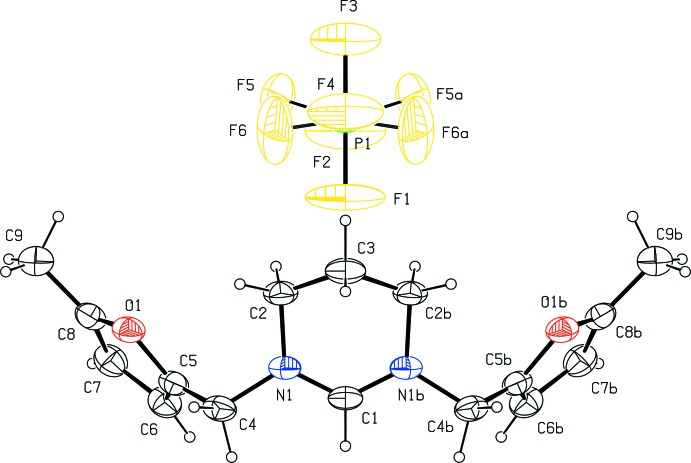
View of the title compound with a crystallographic mirror symmetry. Displacement ellipsoids are shown at the 20% probability levels. The disordered F5, F6 and F5a, F5b atoms are related to each other with a mirror symmetry operation. Symmetry codes: (a) x, 1/2 - y, z; (b) x, 1/2 - y, z.

**Fig. 2.**
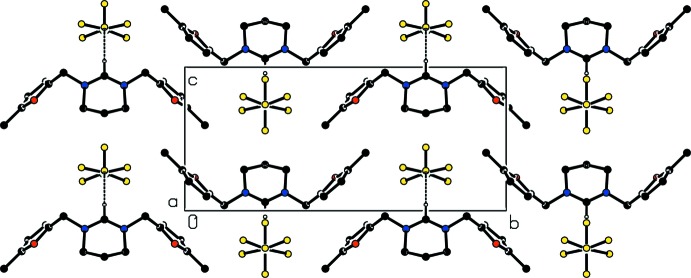
View of the crystal packing and the hydrogen bonding of (I) along a axis. H atoms not involved in hydrogen bonding are omitted for clarity.

**Fig. 3.**
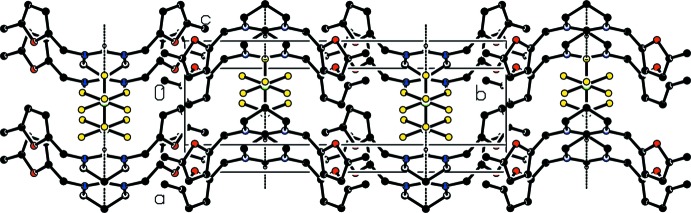
View of the crystal packing and the hydrogen bonding of (I) along c axis. H atoms not involved in hydrogen bonding are omitted for clarity.

## supplementary crystallographic information

### Comment

1,3-Di(5-methylfurfuryl)pyrimidinium hexafluorophosphate salt is conventional N-heterocyclic carbene (NHC) precursors. N-heterocyclic carbenes (NHCs) are generally considered as analogues of phosphine ligands because of their good s-donating but very weak *p*-accepting ability (Arduengo *et al.*, 1992). Normally, NHC-metal complexes have higher stability toward heat, moisture, and oxygen than phosphine metal based complexes, which makes them quite attractive as phosphine substitute. In recent years, NHCs are fully used as organocatalysts and ancillary ligands in transition metal catalyzed reactions (Hermann, 2002). They have attracted many attention, particularly for its functions in catalytic reactions (Akkoç & Gök, 2013; Lee *et al.*, 2013; Bagherzadeh *et al.*, 2012; Çetinkaya *et al.*, 1997; Özdemir *et al.*, 2001; Saba *et al.*, 1991; Yiğit *et al.*, 2007).

Fig. 1 shows the whole molecule (I) whose anions and cations form two parts with a crystallographic mirror symmetry. The C1–N1–C2–C3, C1–N1–C4–C5, C2–N1–C4–C5, N1–C4–C5–O1 and N1–C4–C5–C6 torsion angles are -25.1 (6), -121.5 (4), 60.4 (5), -85.1 (4) and 96.4 (6) °, respectively. All bond lengths of (I) are within *normal* values (Allen *et al.*, 1987).

The crystal structure is stabilized by C—H···F interactions between the anions and cations of (I), forming a three dimensional network (Table 1, Figures 2 and 3).

### Experimental

The reaction for the preparation of heterocyclic salt containing furan moiety was carried out under argon in flame dried glassware using standard Schlenk type flasks. The synthesis of salt containing furan moiety was achieved by the reaction of *N*,*N*'-dialkylpropane-1,3-diamine (1.0 mmol) with ammonium hexafluorophosphate (1.0 mmol) in triethyl orthoformate (5 ml) (Scheme 1). The reaction mixture was heated for 12 h at 353 K. A white solid was precipitated. The precipitation was then crystallized from Et_2_O/EtOH (2:1) at room temperature. The resulting 1,3-di(5-methylfurfuryl)pyrimidinium hexafluorophosphate salt was obtained in good yield.

### Refinement

All H atoms were positioned geometrically and refined by using a riding model, with C—H = 0.93 - 0.97 Å and *U*_iso_(H) = 1.2 or 1.5*U*_eq_(C). The one fluoride atom of the hexafluorophosphate group are disordered over two sites as F5 and F6 in Fig. 1, with equal occupancies of 0.5. Mirror symmetry transformation generates the other equivalent disordered atoms (F5a and F6a in Fig. 1).

### Crystal data

**Table:**

C_16_H_21_N_2_O_2_^+^·PF_6_^−^	*F*(000) = 432
*M**_r_* = 418.32	*D*_x_ = 1.437 Mg m^−^^3^
Monoclinic, *P*2_1_/*m*	Mo *K*α radiation, λ = 0.71073 Å
Hall symbol: -P 2yb	Cell parameters from 1100 reflections
*a* = 6.0793 (6) Å	θ = 2.5–25°
*b* = 18.879 (2) Å	µ = 0.21 mm^−^^1^
*c* = 8.5750 (9) Å	*T* = 296 K
β = 100.747 (5)°	Prism, colourless
*V* = 966.90 (17) Å^3^	0.35 × 0.28 × 0.23 mm
*Z* = 2	

### Data collection

**Table:**

Bruker Kappa APEXII CCD diffractometer	1677 independent reflections
Radiation source: fine-focus sealed tube	1089 reflections with *I* > 2σ(*I*)
Graphite monochromator	*R*_int_ = 0.000
ω scans	θ_max_ = 24.7°, θ_min_ = 2.2°
Absorption correction: part of the refinement model (Δ*F*) (*XABS2*; Parkin *et al.*, 1995)	*h* = −7→7
*T*_min_ = 0.932, *T*_max_ = 0.953	*k* = 0→22
1677 measured reflections	*l* = 0→10

### Refinement

**Table:**

Refinement on *F*^2^	Primary atom site location: structure-invariant direct methods
Least-squares matrix: full	Secondary atom site location: difference Fourier map
*R*[*F*^2^ > 2σ(*F*^2^)] = 0.077	Hydrogen site location: inferred from neighbouring sites
*wR*(*F*^2^) = 0.247	H-atom parameters constrained
*S* = 1.08	*w* = 1/[σ^2^(*F*_o_^2^) + (0.1389*P*)^2^ + 0.421*P*] where *P* = (*F*_o_^2^ + 2*F*_c_^2^)/3
1677 reflections	(Δ/σ)_max_ < 0.001
144 parameters	Δρ_max_ = 0.36 e Å^−^^3^
0 restraints	Δρ_min_ = −0.55 e Å^−^^3^

### Special details

**Table:**

Geometry. Bond distances, angles *etc*. have been calculated using the rounded fractional coordinates. All su's are estimated from the variances of the (full) variance-covariance matrix. The cell e.s.d.'s are taken into account in the estimation of distances, angles and torsion angles
Refinement. Refinement on *F*^2^ for ALL reflections except those flagged by the user for potential systematic errors. Weighted *R*-factors *wR* and all goodnesses of fit *S* are based on *F*^2^, conventional *R*-factors *R* are based on *F*, with *F* set to zero for negative *F*^2^. The observed criterion of *F*^2^ > σ(*F*^2^) is used only for calculating -*R*-factor-obs *etc*. and is not relevant to the choice of reflections for refinement. *R*-factors based on *F*^2^ are statistically about twice as large as those based on *F*, and *R*-factors based on ALL data will be even larger.

### Fractional atomic coordinates and isotropic or equivalent isotropic displacement parameters (Å^2^)

**Table:**

	*x*	*y*	*z*	*U*_iso_*/*U*_eq_	Occ. (<1)
O1	0.0509 (5)	0.46898 (16)	0.2298 (3)	0.0808 (11)	
N1	−0.1021 (5)	0.31127 (19)	0.1262 (4)	0.0706 (11)	
C1	−0.0850 (8)	0.25000	0.0606 (6)	0.069 (2)	
C2	−0.1511 (9)	0.3154 (3)	0.2862 (5)	0.0872 (17)	
C3	−0.2709 (14)	0.25000	0.3206 (9)	0.106 (3)	
C4	−0.0792 (7)	0.3771 (2)	0.0383 (5)	0.0817 (16)	
C5	0.1016 (7)	0.4235 (2)	0.1168 (5)	0.0725 (16)	
C6	0.3108 (8)	0.4325 (3)	0.1001 (6)	0.091 (2)	
C7	0.4019 (9)	0.4871 (3)	0.2040 (6)	0.094 (2)	
C8	0.2431 (9)	0.5075 (3)	0.2828 (5)	0.0852 (17)	
C9	0.2247 (12)	0.5625 (3)	0.4039 (7)	0.120 (3)	
P1	0.3506 (2)	0.25000	0.73427 (19)	0.0793 (7)	
F1	0.3265 (10)	0.25000	0.5584 (6)	0.247 (6)	
F2	0.0993 (9)	0.25000	0.7207 (8)	0.236 (6)	
F3	0.3704 (10)	0.25000	0.9155 (7)	0.218 (5)	
F4	0.6000 (9)	0.25000	0.7399 (8)	0.260 (7)	
F5	0.449 (3)	0.3183 (5)	0.7947 (15)	0.204 (7)	0.500
F6	0.258 (3)	0.3213 (5)	0.699 (2)	0.260 (8)	0.500
H1	−0.05810	0.25000	−0.04260	0.0820*	
H2A	−0.24330	0.35660	0.29500	0.1050*	
H2B	−0.01270	0.32020	0.36270	0.1050*	
H3A	−0.28490	0.25000	0.43150	0.1280*	
H3B	−0.42050	0.25000	0.25680	0.1280*	
H4A	−0.05180	0.36490	−0.06630	0.0980*	
H4B	−0.21940	0.40290	0.02440	0.0980*	
H6	0.38470	0.40730	0.03220	0.1090*	
H7	0.54600	0.50550	0.21560	0.1130*	
H9A	0.36010	0.58990	0.42480	0.1800*	
H9B	0.20190	0.54010	0.50010	0.1800*	
H9C	0.10040	0.59310	0.36490	0.1800*	

### Atomic displacement parameters (Å^2^)

**Table:**

	*U*^11^	*U*^22^	*U*^33^	*U*^12^	*U*^13^	*U*^23^
O1	0.0858 (19)	0.098 (2)	0.0648 (18)	0.0177 (16)	0.0303 (14)	0.0102 (15)
N1	0.070 (2)	0.094 (2)	0.0496 (19)	0.0089 (16)	0.0162 (14)	0.0031 (17)
C1	0.050 (3)	0.108 (5)	0.048 (3)	0.0000	0.008 (2)	0.0000
C2	0.104 (3)	0.107 (3)	0.057 (3)	0.013 (3)	0.032 (2)	0.000 (2)
C3	0.113 (5)	0.148 (7)	0.071 (4)	0.0000	0.052 (4)	0.0000
C4	0.085 (3)	0.102 (3)	0.058 (2)	0.027 (2)	0.013 (2)	0.014 (2)
C5	0.085 (3)	0.080 (3)	0.057 (2)	0.025 (2)	0.025 (2)	0.0179 (19)
C6	0.088 (3)	0.101 (4)	0.094 (4)	0.021 (2)	0.042 (3)	0.017 (3)
C7	0.087 (3)	0.102 (4)	0.098 (4)	−0.003 (3)	0.031 (3)	0.018 (3)
C8	0.105 (3)	0.086 (3)	0.067 (3)	0.005 (3)	0.022 (2)	0.020 (2)
C9	0.170 (6)	0.112 (4)	0.083 (4)	−0.004 (4)	0.039 (4)	0.000 (3)
P1	0.0552 (9)	0.1200 (15)	0.0645 (11)	0.0000	0.0157 (7)	0.0000
F1	0.104 (4)	0.578 (18)	0.065 (3)	0.0000	0.030 (3)	0.0000
F2	0.077 (3)	0.516 (17)	0.121 (5)	0.0000	0.036 (3)	0.0000
F3	0.111 (4)	0.449 (14)	0.094 (4)	0.0000	0.023 (3)	0.0000
F4	0.071 (3)	0.59 (2)	0.116 (5)	0.0000	0.012 (3)	0.0000
F5	0.291 (15)	0.108 (6)	0.199 (11)	−0.077 (8)	0.013 (10)	−0.036 (6)
F6	0.242 (13)	0.114 (7)	0.36 (2)	0.065 (7)	−0.111 (14)	0.018 (9)

### Geometric parameters (Å, º)

**Table:**

P1—F6^i^	1.469 (11)	C5—C6	1.317 (7)
P1—F1	1.488 (5)	C6—C7	1.407 (8)
P1—F2	1.510 (6)	C7—C8	1.334 (8)
P1—F3	1.536 (6)	C8—C9	1.487 (8)
P1—F4	1.508 (6)	C1—H1	0.9300
P1—F5	1.474 (11)	C2—H2B	0.9700
P1—F6	1.469 (11)	C2—H2A	0.9700
P1—F5^i^	1.474 (11)	C3—H3A	0.9700
F5—F6	1.29 (2)	C3—H3B	0.9700
O1—C5	1.372 (5)	C4—H4A	0.9700
O1—C8	1.379 (6)	C4—H4B	0.9700
N1—C2	1.459 (6)	C6—H6	0.9300
N1—C4	1.474 (5)	C7—H7	0.9300
N1—C1	1.299 (4)	C9—H9C	0.9600
C2—C3	1.491 (7)	C9—H9A	0.9600
C4—C5	1.467 (6)	C9—H9B	0.9600
			
F4—P1—F5^i^	69.5 (7)	N1—C4—C5	113.8 (3)
F4—P1—F6^i^	110.6 (7)	O1—C5—C6	110.2 (4)
F5—P1—F6	52.1 (9)	C4—C5—C6	133.1 (4)
F5—P1—F5^i^	122.0 (8)	O1—C5—C4	116.7 (4)
F5—P1—F6^i^	171.5 (8)	C5—C6—C7	107.4 (5)
F5^i^—P1—F6	171.5 (8)	C6—C7—C8	107.2 (5)
F6—P1—F6^i^	132.9 (9)	O1—C8—C9	115.4 (5)
F5^i^—P1—F6^i^	52.1 (9)	O1—C8—C7	109.2 (4)
F2—P1—F5^i^	111.4 (7)	C7—C8—C9	135.3 (6)
F2—P1—F6^i^	69.0 (7)	N1^i^—C1—H1	117.00
F3—P1—F4	94.5 (4)	N1—C1—H1	117.00
F3—P1—F5	72.2 (5)	N1—C2—H2A	110.00
F1—P1—F2	90.9 (4)	C3—C2—H2B	110.00
F1—P1—F3	178.9 (3)	N1—C2—H2B	110.00
F1—P1—F4	86.6 (4)	C3—C2—H2A	110.00
F1—P1—F5	108.3 (5)	H2A—C2—H2B	108.00
F1—P1—F6	80.2 (7)	C2^i^—C3—H3A	109.00
F1—P1—F5^i^	108.3 (5)	C2—C3—H3A	109.00
F1—P1—F6^i^	80.2 (7)	C2—C3—H3B	109.00
F2—P1—F3	88.0 (4)	H3A—C3—H3B	108.00
F2—P1—F4	177.5 (4)	C2^i^—C3—H3B	109.00
F2—P1—F5	111.4 (7)	N1—C4—H4B	109.00
F2—P1—F6	69.0 (7)	N1—C4—H4A	109.00
F4—P1—F5	69.5 (7)	H4A—C4—H4B	108.00
F4—P1—F6	110.6 (7)	C5—C4—H4A	109.00
F3—P1—F5^i^	72.2 (5)	C5—C4—H4B	109.00
F3—P1—F6^i^	99.4 (7)	C5—C6—H6	126.00
F3—P1—F6	99.4 (7)	C7—C6—H6	126.00
P1—F5—F6	63.7 (8)	C8—C7—H7	126.00
P1—F6—F5	64.2 (8)	C6—C7—H7	126.00
C5—O1—C8	105.9 (3)	H9B—C9—H9C	110.00
C1—N1—C2	120.1 (4)	C8—C9—H9A	109.00
C2—N1—C4	119.4 (4)	C8—C9—H9B	110.00
C1—N1—C4	120.5 (4)	C8—C9—H9C	109.00
N1—C1—N1^i^	125.9 (4)	H9A—C9—H9B	109.00
N1—C2—C3	109.6 (4)	H9A—C9—H9C	109.00
C2—C3—C2^i^	111.9 (6)		
			
F5^i^—P1—F5—F6	−172.5 (11)	C2—N1—C1—N1^i^	−1.9 (7)
F1—P1—F6—F5	−122.5 (10)	C1—N1—C4—C5	−121.5 (4)
F2—P1—F6—F5	142.8 (11)	C4—N1—C1—N1^i^	−180.0 (4)
F1—P1—F5—F6	61.1 (11)	C2—N1—C4—C5	60.4 (5)
F2—P1—F5—F6	−37.4 (11)	C1—N1—C2—C3	−25.1 (6)
F3—P1—F5—F6	−117.9 (11)	C4—N1—C2—C3	153.1 (5)
F4—P1—F5—F6	140.1 (11)	N1—C2—C3—C2^i^	51.2 (7)
F3—P1—F6—F5	58.6 (10)	N1—C4—C5—C6	96.4 (6)
F4—P1—F6—F5	−39.9 (11)	N1—C4—C5—O1	−85.1 (4)
F6^i^—P1—F6—F5	170.8 (12)	C4—C5—C6—C7	177.5 (5)
C5—O1—C8—C7	0.9 (5)	O1—C5—C6—C7	−1.1 (6)
C5—O1—C8—C9	178.1 (4)	C5—C6—C7—C8	1.7 (6)
C8—O1—C5—C4	−178.7 (4)	C6—C7—C8—C9	−178.0 (6)
C8—O1—C5—C6	0.2 (5)	C6—C7—C8—O1	−1.6 (6)

Symmetry code: (i) *x*, −*y*+1/2, *z*.

### Hydrogen-bond geometry (Å, º)

**Table:**

*D*—H···*A*	*D*—H	H···*A*	*D*···*A*	*D*—H···*A*
C1—H1···F2^ii^	0.93	2.40	3.312 (8)	167
C1—H1···F4^iii^	0.93	2.52	3.044 (8)	116

Symmetry codes: (ii) *x*, *y*, *z*−1; (iii) *x*−1, *y*, *z*−1.
